# NonClasGP-Pred: robust and efficient prediction of non-classically secreted proteins by integrating subset-specific optimal models of imbalanced data

**DOI:** 10.1099/mgen.0.000483

**Published:** 2020-11-27

**Authors:** Chao Wang, Jin Wu, Lei Xu, Quan Zou

**Affiliations:** ^1^​ Institute of Fundamental and Frontier Sciences, University of Electronic Science and Technology of China, Chengdu, PR China; ^2^​ School of Management, Shenzhen Polytechnic, Shenzhen, PR China; ^3^​ School of Electronic and Communication Engineering, Shenzhen Polytechnic, Shenzhen, PR China; ^4^​ Hainan Key Laboratory for Computational Science and Application, Hainan Normal University, Haikou, PR China

**Keywords:** feature selection, imbalanced dataset, machine learning, model ensemble, non-classically secreted proteins

## Abstract

Non-classically secreted proteins (NCSPs) are proteins that are located in the extracellular environment, although there is a lack of known signal peptides or secretion motifs. They usually perform different biological functions in intracellular and extracellular environments, and several of their biological functions are linked to bacterial virulence and cell defence. Accurate protein localization is essential for all living organisms, however, the performance of existing methods developed for NCSP identification has been unsatisfactory and in particular suffer from data deficiency and possible overfitting problems. Further improvement is desirable, especially to address the lack of informative features and mining subset-specific features in imbalanced datasets. In the present study, a new computational predictor was developed for NCSP prediction of gram-positive bacteria. First, to address the possible prediction bias caused by the data imbalance problem, ten balanced subdatasets were generated for ensemble model construction. Then, the F-score algorithm combined with sequential forward search was used to strengthen the feature representation ability for each of the training subdatasets. Third, the subset-specific optimal feature combination process was adopted to characterize the original data from different aspects, and all subdataset-based models were integrated into a unified model, NonClasGP-Pred, which achieved an excellent performance with an accuracy of 93.23 %, a sensitivity of 100 %, a specificity of 89.01 %, a Matthew’s correlation coefficient of 87.68 % and an area under the curve value of 0.9975 for ten-fold cross-validation. Based on assessment on the independent test dataset, the proposed model outperformed state-of-the-art available toolkits. For availability and implementation, see: http://lab.malab.cn/~wangchao/softwares/NonClasGP/.

## Data Summary

We confirm that all supporting data, code and protocols have been provided within the article or through Supplementary Material.

**Impact Statement**

Non-classically secreted proteins (NCSPs) are proteins that are located in the extracellular environment, although there is a lack of known signal peptides or secretion motifs. NCSP identification remains challenging due to insufficient discernible features and the performance of existing methods has been unsatisfactory. We therefore developed a new computational predictor, NonClasGP-Pred, for NCSP prediction of gram-positive bacteria. This achieved excellent performance with an accuracy of 93.23 %, a sensitivity of 100 %, a specificity of 89.01 %, a Matthew’s correlation coefficient of 87.68 % and an area under the curve value of 0.9975 for ten-fold cross-validation. Based on assessment of the independent test, the proposed model outperformed state-of-the-art available toolkits. NonClasGP-Pred is a useful bioinformatics tool for analyses of NCSPs. It will help to determine the biological function of NCSPs related to bacterial virulence and cell defence.

## Introduction

Secreted proteins can produce a marked effect only when they are transported across the cell membrane to reach their function venue. Generally, secreted proteins are synthesized initially as precursors in the cytoplasm, and they are then targeted toward the translocation machinery and finally delivered into the extracellular space through a proteinaceous channel. The majority of secreted proteins depend on classical Sec- or Tat-dependent secretion pathways [1–3], where the known, predictable signal peptide or secretion motifs in the protein sequence are necessary for the two pathways.

Nevertheless, proteins without any known signal peptides or secretion motifs can also be exported into the extracellular space. As their secretion pathway remains ill-defined, they are termed non-classically secreted proteins (NCSPs) [4–6]. Because NCSPs display different biological functions when they are in the cytoplasm and extracellular space, they were designated as so-called moonlighting proteins, meaning these proteins show functional variety in different pathways [7]. For example, glyceraldehyde 3-phosphate dehydrogenase is essential for the glycolytic pathway in cytoplasm, while it played an important role in plasminogen binding on the bacterial surface [8]. Previous studies have shown that NCSPs can adhere to host epithelia and components, affect cell viability, and modulate host immune responses [5] involving the function of bacterial virulence and cell defence [9]. For heterologous protein production, many bottlenecks were encountered in the classical Sec- or Tat-dependent secretion pathways. Most heterologously expressed proteins are unable to cross the cytoplasmic membrane and they easily form inclusion bodies that are difficult to renature. It is remarkable that NCSPs have been successfully used as export signals to assist in the secretion of specific proteins of interest in biotechnology [10, 11].

Accurate protein localization is essential for all living organisms. In terms of gram-positive bacteria, bioinformatics tools that are available for NCSP prediction were mainly developed based on mammalian proteins. For instance, SecretomeP [12] is the first reported tool that can be used for NCSP identification, but positive data were not composed of real NCSPs but simulated by mammalian classically secreted proteins whose signal part was removed. SecretP [13] was trained on a dataset of proteins in which non-classically secreted gram-positive bacterial proteins were deficient; NClassG+ [14] and the latest PeNGaRoo [15] provided a more reasonable result when tested on an independent set. Although previous studies have greatly contributed to the discovery of NCSPs, further improvement is desirable, especially to address the lack of informative features and mining subset-specific features for imbalanced datasets.

The purpose of the present study was to develop a new computational predictor to further improve the performance of the NCSP prediction of gram-positive bacteria. First, to address the possible prediction bias caused by the data imbalance problem, ten balanced subdatasets were generated for ensemble model construction. Second, ten feature descriptors were used to encode the protein sequences into numerical vectors, and the F-score algorithm combined with sequential forward search was applied to reinforce the feature representation ability for each of the training subdatasets. Third, the subset-specific optimal feature combination process was adopted to characterize the original data from different aspects. Finally, all subdataset-based models were integrated to improve the generalizability of the model. When assessed based on the independent test, the proposed ensemble model achieved superior predictive performance and outperformed state-of-the-art available toolkits.

## Methods

### Datasets

In this study, we adopted the benchmark datasets constructed by Zhang *et al*. [15] to specifically identify NCSPs of gram-positive bacteria. We used these datasets for the following reasons. First, the proteins of the positive dataset, NCSPs of gram-positive bacterial proteins, were experimentally verified, and each protein was confirmed by at least three different research groups in at least three different bacterial species [5, 15]. Second, the sequence identity was reduced to 80 % to avoid potential redundancy. For the negative dataset, 1084 proteins that localized in the cytoplasm [7, 15] were used in this work. Similar, the sequence identity was reduced to 80 % to avoid potential redundancy. The final training dataset contained 141 positive and 446 negative protein samples. To address this data imbalance issue, we generated ten balanced datasets, termed TD1, TD2, …, and TD10, each of which comprised all the 141 positive samples and an equal number of negative samples that were randomly chosen from the negative dataset.

An independent test dataset containing 34 positive samples and 34 negative samples was used for further performance evaluation and comparison. For more details regarding the benchmark datasets, see Zhang *et al*. [15].

### Feature extraction

To build an accurate and reliable bioinformatics tool, sufficient feature information should be incorporated into the model [16–19]. In this study, ten feature-encoding algorithms were used to represent the protein sequence, including amino acid composition (AAC), composition of k-spaced amino acid pairs (CKSAAP), dipeptide composition (DPC), dipeptide deviation from expected mean (DDE), composition (CTDC), transition (CTDT), conjoint triad (CTriad), quasi-sequence-order (QSOrder), normalized Moreau-Broto (NMBroto) and pseudoamino acid composition (PAAC). They were categorized into three groups, i.e. amino acid composition group, amino acid distribution group and sequence order group. The above feature-encoding algorithms are described in detail in the Supplementary methods, and a brief introduction of these algorithms is provided below.

#### Amino acid composition-based features

The AAC descriptor [20, 21] encodes the frequencies of all 20 amino acids in a protein sequence. The CKSAAP descriptor [22] measures the frequency of any k residue-spaced amino acid pairs. The DPC and DDE [23] calculate the frequencies of all dipeptides.

#### Amino acid distribution-based features

The composition (CTDC) and transition (CTDT) features [24] characterize the amino acid distribution patterns or physicochemical properties in a protein. Twenty amino acids are categorized into three groups according to their physicochemical properties. The composition descriptor represents the percentage of each group of residues in the protein sequence. The transition descriptor characterizes the frequencies of three kinds of residue pairs. Thirteen types of physicochemical property (Table S1, available in the online version of this article) are used to compute the features of CTDC and CTDT. CTriad [25] characterizes the properties of one amino acid and its neighbours, where any three continuous amino acids are regarded as a single unit.

#### Sequence order-based features

The QSOrder features characterize the sequence order based on the Schneider–Wrede physicochemical distance matrix [26] and the Grantham chemical distance matrix [27]. The NMBroto descriptor [28] is used to characterize the distribution of amino acid properties along the sequence. In this paper, eight amino acid indices are selected from the AAindex database (Table S2). PAAC introduces a discrete model derived from the amino acid sequence to represent its sequence order or pattern information [29, 30].

### Framework of NonClasGP-Pred

**Fig. 1.**
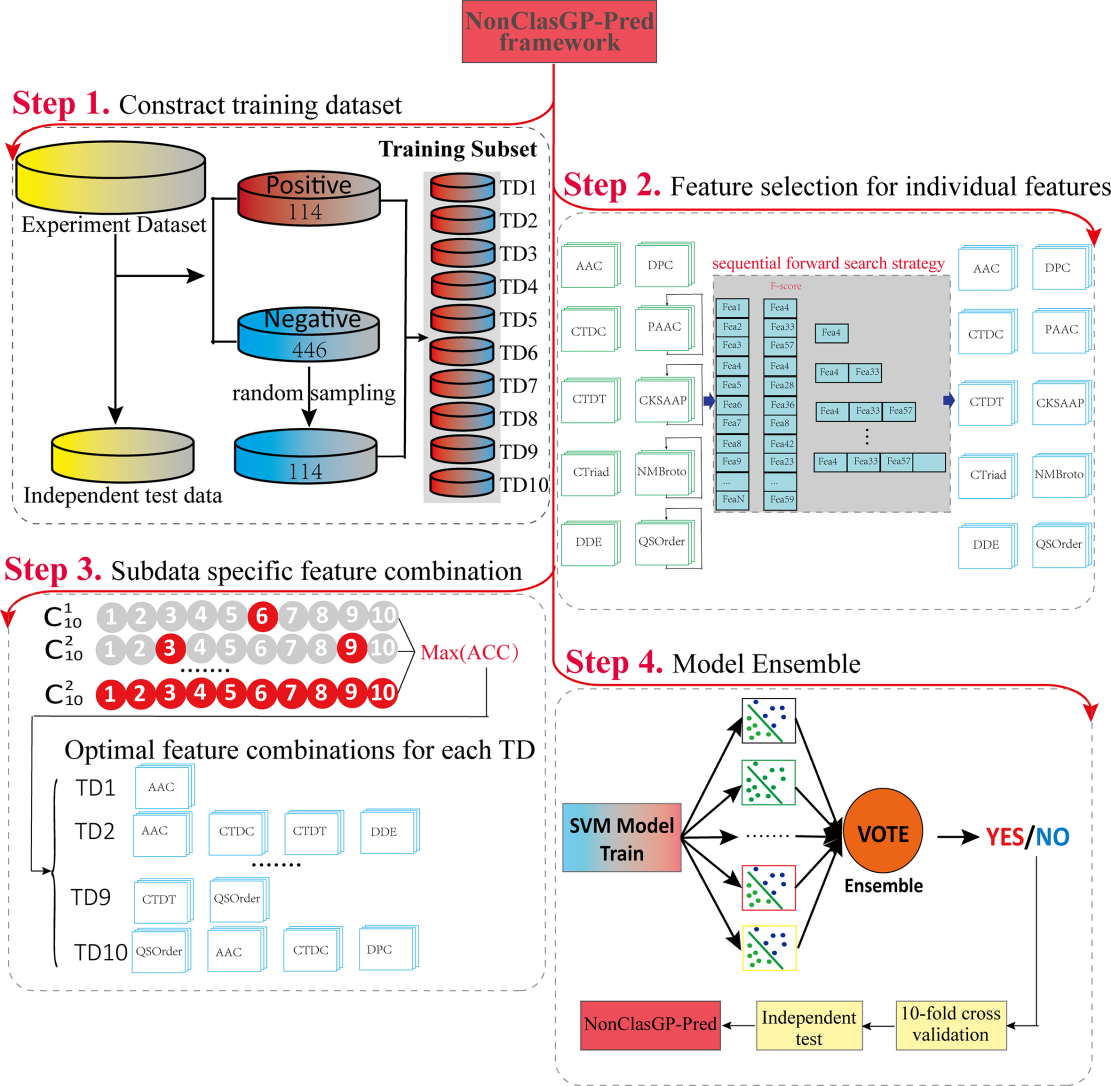
Framework of NonClasGP-Pred.

Fig. 1 illustrates the framework of NonClasGP-Pred, which involves four main steps: (i) feature encoding, (ii) feature selection, (iii) feature combination, and (iv) model ensemble and evaluation. The feature-encoding methods are presented in the feature extraction section above, and the remaining three procedures are described in detail below.

#### Parameter optimization for individual feature descriptor

The feature dimensions of four of the ten feature descriptors are determined by the parameter of the related algorithms (Table 1). To achieve the best performance on each individual descriptor, the four parameters were optimized, and the search range for each of them is listed in Table 1, where ten-fold cross validation was used to measure the performance of the model with different parameters.

**Table 1.** Descriptor feature dimensions and parameter search range

Feature descriptor	Parameter	Feature dimension	Search range	Optimal value
PAAC	λ	λ+20	[1, 2, 3, …, 50]	11
CKSAAP	K	(K+1)*400	[0,1, 2, 3, …, 9]	9
NMBroto	nlag	nlag*8	[1, 2, 3, …, 50]	19
QSOrder	nlag	nlag*2+40	[1, 2, 3, …, 50]	4
AAC	–	20	–	–
CTDC	–	39	–	–
CTDT	–	39	–	–
CTriad	–	343	–	–
DDE	–	400	–	–
DPC	–	400	–	–

#### Feature selection for individual features

To include sufficient information, we used all the feature encodings as described above. High-dimensional features are often redundant and noisy, which affects model generalization, and they are computationally expensive [31, 32]. A feature selection procedure was performed to identify the most discriminative features by removing the redundant and irrelevant features. In this study, a two-step feature selection method was processed to choose the optimal subsets. First, the F-score method was applied to compute the feature importances and sort them in descending order, and the F-score value of the *i*th feature is calculated as below. After that, the optimal feature subsets were selected using the sequential forward search (SFS) method. In SFS, we added features from the sorted list one by one to train and evaluate the prediction model by ten-fold cross validation (for more details, see Model construction and evaluation below). Last, the feature subsets leading to the model with the highest accuracy (ACC) are extracted as the optimal features.

$$F-score(i)=\frac{({\overset{-}{x}}_{i}^{\left(+\right)}-{\overset{-}{x}}_{i}{)}^{2}+({\overset{-}{x}}_{i}{)}^{\left(-\right)}-{\overset{-}{x}}_{i}{)}^{2}}{\frac{1}{n^{+}-1}\sum_{k=1}^{n+}(x_{k,i}^{\left(+\right)}-{\overset{-}{x}}_{i}^{\left(+\right)})2+\frac{1}{n^{-}-1}\sum_{k=1}^{n-}(x_{k,i}^{\left(-\right)}-{\overset{-}{x}}_{i}^{\left(-\right)}{)}^{2}}$$

where *n* ^*+*^ is the number of positive sample, *n* ^*−*^ is the number of negative samples; ${\overset{-}{x}}_{i}$ represents the feature average value of the *i*th feature, ${{\overset{-}{x}}_{i}}^{\left(+\right)}$ is the average value of the *i*th feature in the positive sample, ${{\overset{-}{x}}_{i}}^{\left(-\right)}$ is the average value of the *i*th feature in the negative sample; $x_{k,i}^{\left(+\right)}$ and $x_{k,i}^{\left(-\right)}$ represent the *i*th feature of the *k*th positive and negative samples, respectively. A feature that has superior discrimination ability is correlated to a high F-score.

#### Feature combination

To build a robust prediction model with good performance, we not only individually used the ten types of optimal feature subsets as described in the previous section but also any combination among the ten feature subsets using an exhaustive searching. We evaluated all possible 1023 models ($c_{10}^{1},c_{10}^{2},c_{10}^{3},\cdot \cdot \cdot ,c_{10}^{10}$) for each of the ten training datasets. We note that the results of Zhang *et al*. [15] might be overfitted as it resulted in an accuracy of 0.900 in the training dataset and an accuracy of 0.779 in the independent test data. Our preliminary experiment results (Table S3) also showed similar overfitting problems. On this basis, for each of the 1023 models, the independent test data instead of the *n*-fold cross validation method was employed for best model selection. More specifically, taking the combination NMBroto, QSOrder, CTDT and CTriad in TD4 as an example, the four feature subsets were combined first, which resulting a 374-D vector. The model was trained on the combined training dataset, and then it was tested on the independent test dataset based on the ACC metric. Note that the independent test dataset was not involved in any model construction process.

### Model construction and evaluation

In this study, the powerful support vector machine (SVM) algorithm is employed to train our binary classification model, and this method has been extensively used in several bioinformatics fields [33–41], such as disease genes [42, 43] and non-coding RNAs [44, 45]. We implemented SVM with the Python package in scikit-learn (v 0.22.1). Two critical parameters, namely the kernel parameter γ and the penalty parameter C, were optimized by the grid search approach. The radial basis function (RBF) was used as the kernel function of SVM, and the search range for C and γ is [0.01, 0.05, 0.1, 0, 1, 5, …, 90, 95, 100] and [0.0001, 0.0002, 0.0004, 0.0006, 0.0008, …, 2, 4, 6, 8], respectively.

To improve the performance of the NonClasGP model, an ensemble learning model was built in this study, which used majority voting to integrate the prediction results of the above ten individual models, each of which was built on the optimal feature combinations. The performance of the ensemble model NonClasGP-Pred was evaluated by five commonly used metrics [15, 40, 46–59]: ACC, specificity (SP), sensitivity (SN), Matthews correlation coefficient (MCC) and AUC. They are calculated as follows:

(1) $$ACC=\frac{TP+TN}{TP+TN+FP+FN}$$

(2) $$SN=\frac{TP}{TP+FP}$$

(3) $$SP=\frac{TN}{TN+FP}$$

(4) $$MCC=\frac{TP\times TN-FP\times FN}{\sqrt{\left(FP+TP)(FN+TP)(FP+TN)(FN+TN\right)}}$$

(5) $$TPR=\frac{TP}{TP+FN}$$

(6) $$FPR=\frac{FP}{TN+FP}$$

The metric AUC represents the area under the receiver operating characteristic (ROC) curve, which is calculated by the false positive rate (FPR) and the true positive rate (TPR) under various thresholds; the TPR and the FPR are calculated as Equation (5) and (6), respectively.

where TP=true positive, FP=false positive, TN=true negative and FN=false negative.

Of the five metrics, SN and SP are used to evaluate the model performance with respect to the positive samples and negative samples, respectively, and the remaining three metrics are global prediction performance indicators. Moreover, ten-fold cross validation was used for evaluation of model performance.

## Results and Discussion

### Descriptor parameter optimization

**Fig. 2.**
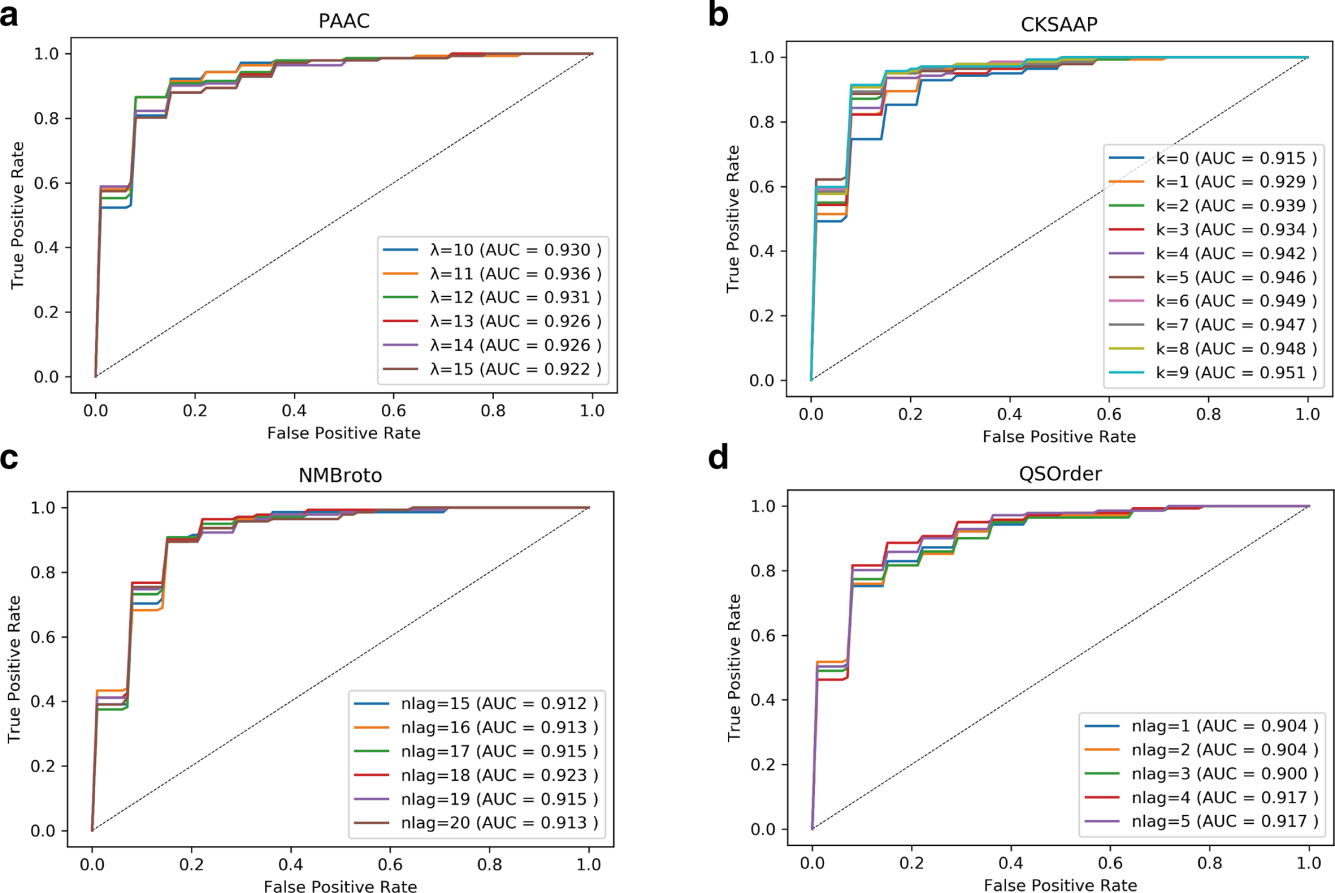
ROC curve of four feature descriptors with different algorithm parameters.

As shown in Table 1, the feature vector dimensions of four descriptors, including PAAC, CKSAAP, NMBroto and QSOrder, depended on the parameter value of the algorithm. To make each of the descriptors as informative as possible, the parameters were preoptimized before the feature selection procedure. Note that for computational convenience and to adequately represent the balanced training dataset, the parameter optimization process was only subjected to TD1 whose optimal parameters were applied to other 9 datasets. The ROC curves around the highest parameters are shown in Fig. 2. The PAAC achieved the best performance when the value of parameter λ was 11 (Fig. 2a); CKSAAP resulted in the highest AUC value of 0.951 when the parameter k was 9 (Fig. 2b); the maximum AUC value of NMBroto and QSOrder was obtained when the parameter nlag was set to 19 and 4, respectively (Fig. 2c, d). The feature vector dimensions of the four optimized descriptors and the other size-fixed descriptors are presented in Table 1.

### Feature selection for individual features

**Fig. 3.**
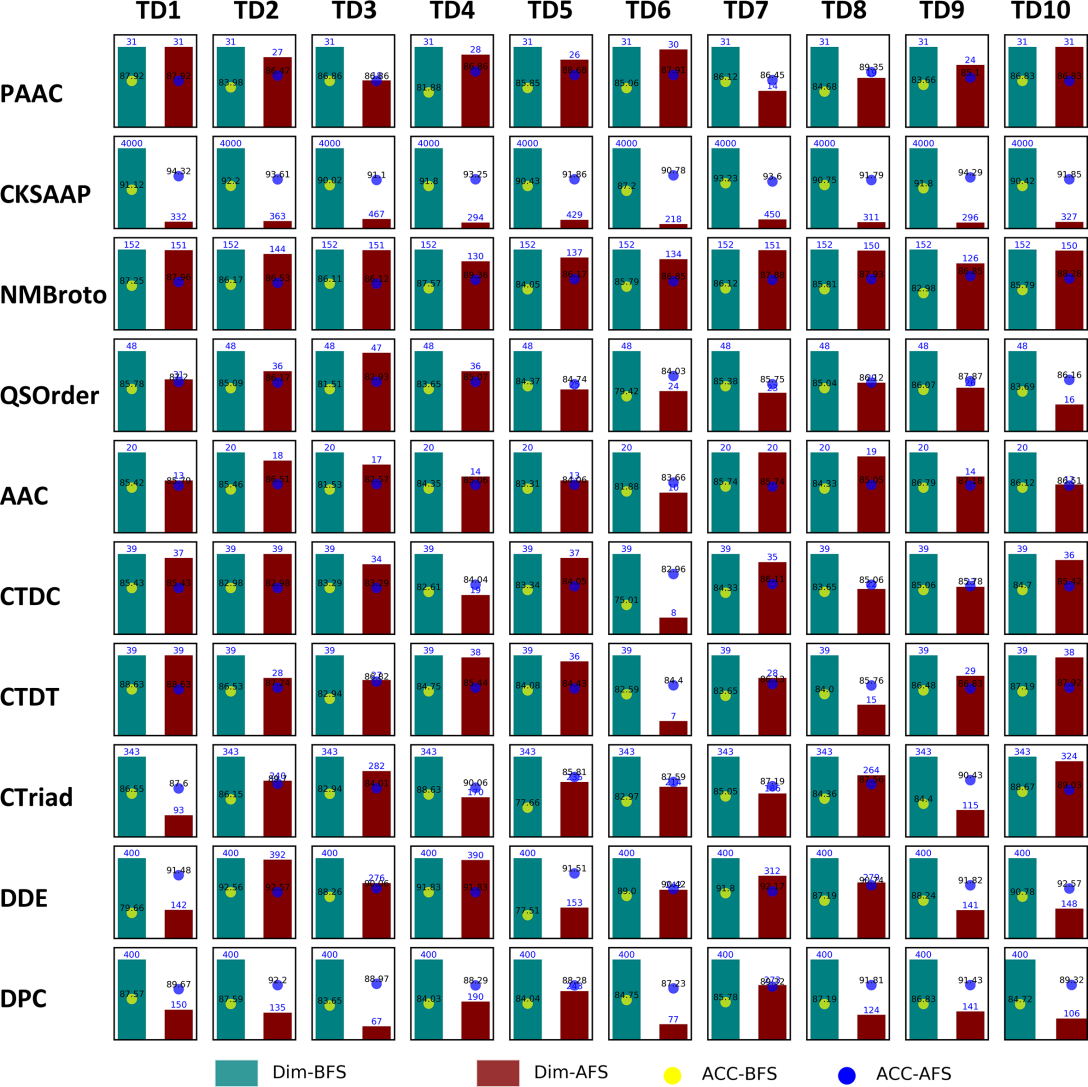
Feature dimension and model performance (ACC) before and after feature selection. Dim-BFS: feature dimension before feature selection, Dim-AFS: feature dimension after feature selection, ACC-BFS: ACC of model before feature selection, and ACC-AFS: ACC of model after feature selection.

As described in the Methods, the F-score and SFS were used for feature selection. We performed this procedure on the ten balanced training datasets, TD1, TD2, …, TD10, independently. The results of feature selection are illustrated in Fig. 3. The dimensions for the majority of features were reduced, especially for those with higher dimensionality, as they tend to contain more redundant information, such as features in CKSAAP, DDE and DPC. For a specific descriptor, the dimensions of the optimal feature subset among different training datasets were also different. For instance, the optimized dimension of AAC feature ranged from 14 to 31, and that of the DPC varied from 77 to 243. This indicates that the information embodied in different TDs is inconsistent to some extent. Furthermore, the performance of the model trained on the optimal feature subset was improved in terms of the metric ACC, demonstrating that the feature selection strategy is beneficial for improving the feature representation ability and contributes to improving model performance.

### Combination of various features to optimize the prediction model

**Fig. 4.**
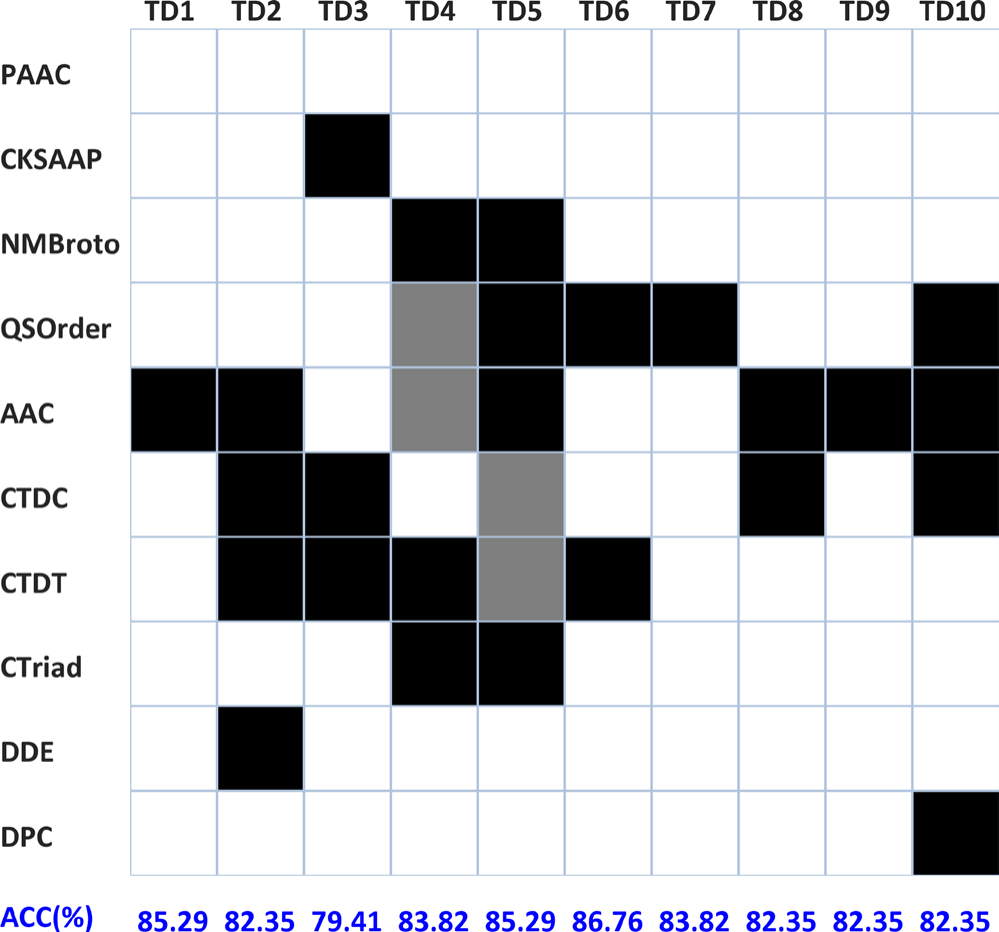
Subdataset-specific optimal feature combination. The black squares represent the composition of the best feature combination for a specific training subdataset based on the metric ACC, and the grey squares represents the alternative feature of the best model. For instance, QSOrder and AAC are alternatives for each other for the optimal feature subset of TD4; in other words, the combination of NMBroto + QSOrder + CTDT + CTriad achieved an ACC value equal to that of the combination of NMBroto + AAC + CTDT + CTriad.

To construct the optimal prediction model, we investigated all possible combinations of the ten feature subsets obtained in the above section. For each training dataset, 1023 models were constructed in total, and these models were then evaluated on independent test data to avoid the overfitting problem based on the ACC metric. Fig. 4 shows the best feature combinations for each of the ten training datasets. As shown in Fig. 4, the best feature combinations of the ten training datasets are different from each other. For instance, the optimal models of TD1 and TD9 are built only on AAC, and the best models for TD8 and TD6 are based on two features, while that of TD5 is constructed based on five features (NMBroto, QSOrder, ACC, CTriad and CTDC/CTDT).

With regard to the versatility of feature encodings, AAC is the most commonly used, as it is adopted by seven of the ten best models. CTDT, CTDC and QSOrder are included in five of the ten best models. Two feature representation strategies, CTriad and NMBroto, are informative for two of the ten models, indicating that these feature descriptors are more predictive and discriminative than the others. Additionally, some feature encodings are specific for certain training data. For example, three of the encodings, DPC, DDE and CKSAAP, are only used by one of the ten models, demonstrating that these features can probably capture some specific characteristics. However, although PAAC achieved a high AUC score in the training data (Fig. 2a), it was absent from all ten optimal models when evaluated on the independent dataset, suggesting that PAAC-encoded features lack generalization ability. In terms of model performances, the ACC value of the model trained on different training data is also not the same, where the maximum ACC value (86.76 %) was achieved on TD6, and the minimum ACC value (79.41 %) was obtained on TD3.

As described above, it can be concluded that each training subset represents only a part of the information from the complete dataset. Therefore, integrating the optimal models above would be helpful to improve the performance of the predictive models.

### Improving model performance by ensemble learning

**Fig. 5.**
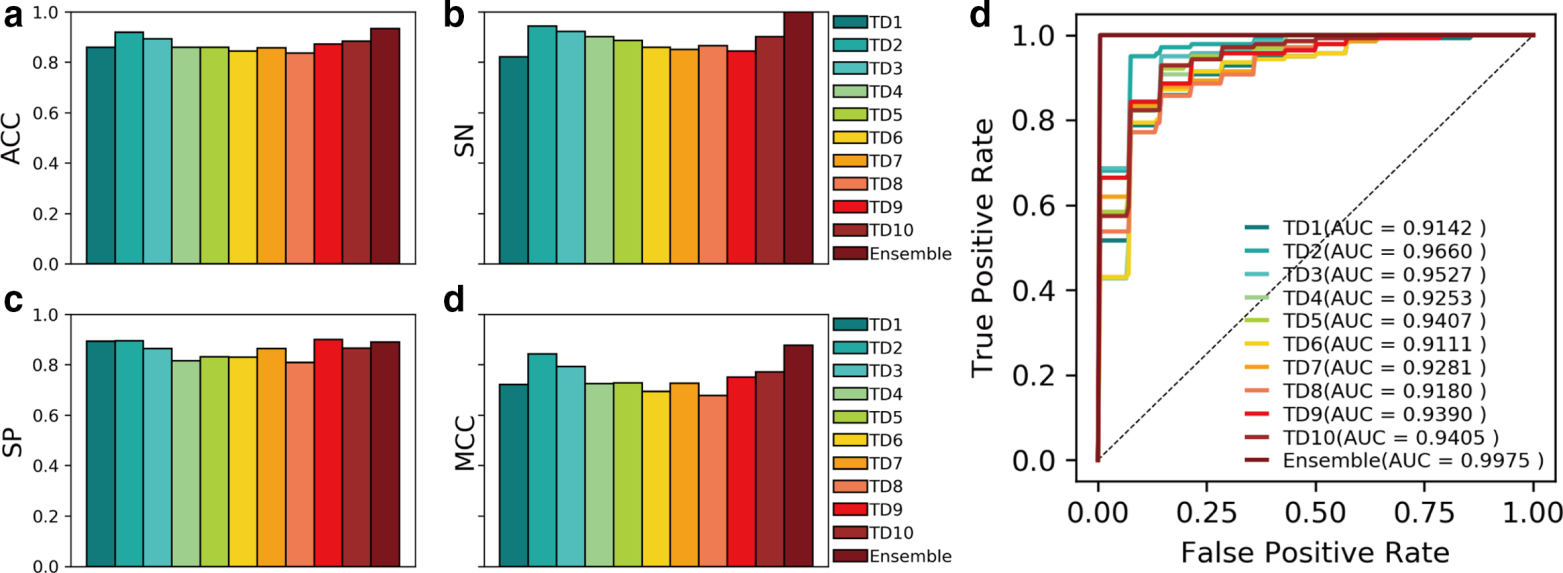
Performance comparison between the models built on individual training subsets and the ensemble model by ten-fold cross validation.

To improve the performance of the NonClasGP-Pred model, an ensemble learning model was built to integrate all the subset-specific optimal models as mentioned above. To intuitively exhibit the effectiveness of the ensemble strategy, we plotted the ten-fold cross validation results (Table S4) of the ten individual models and the ensemble NonClasGP-Pred model in Fig. 5. It can be clearly seen that the ensemble model achieved better performance than the individual models in ACC, SN, MCC and AUC. Specifically, the NonClasGP-Pred model achieved the best ACC of 93.23 % (Fig. 5a), which resulted in an average improvement of 6.45 % compared with the remaining individual models. Similar results can also be seen for SN and MCC, whereas the average values were increased by 12.12 and 13.41 % (Fig. 5b, d), respectively. The SP value achieved by the ensemble model is the fourth best (89.01 %), which is slightly lower than that of TD9 (90.00 %), TD1 (89.38 %) and TD2 (89.28 %) (Fig. 5c). Notably, the ensemble model enhances the AUC by 3.15–8.64 % (Fig. 5e), indicating that the ensemble strategy is capable of effectively improving the model performance.

### Comparison of NonClasGP-Pred with existing predictors

**Fig. 6.**
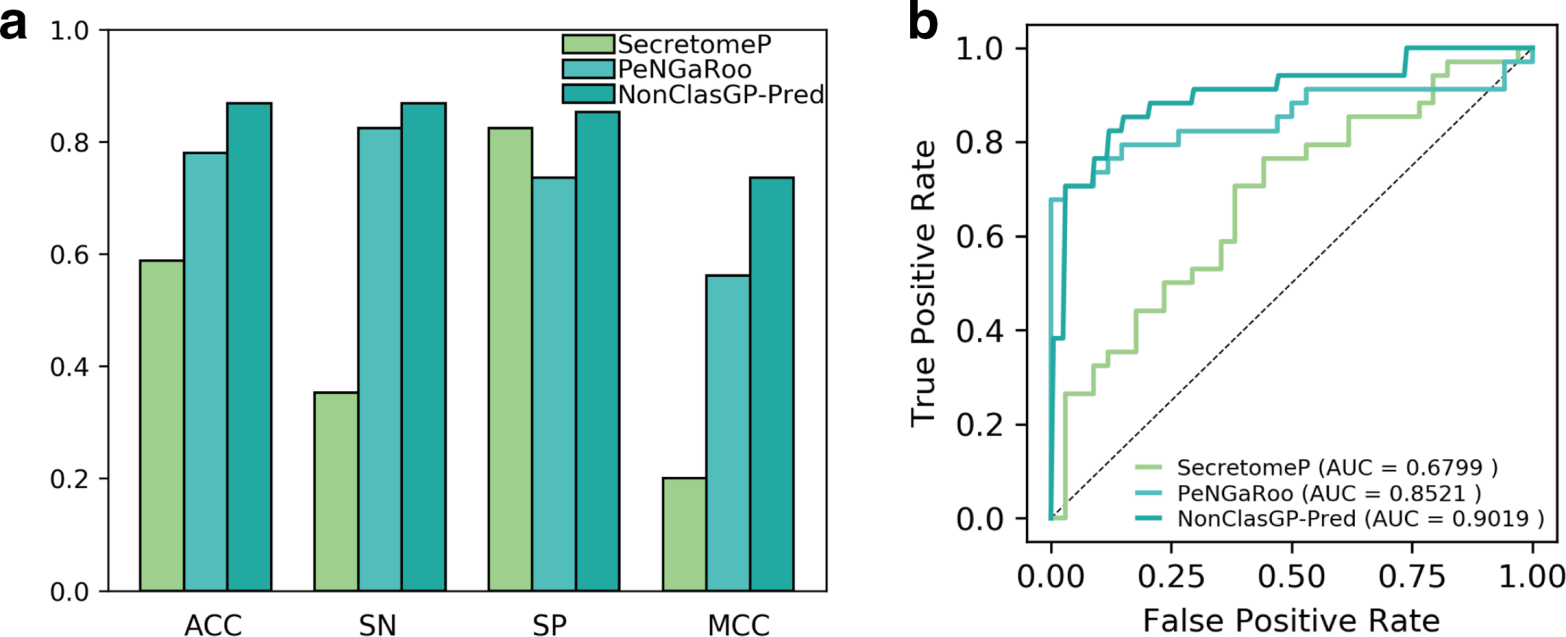
Performance comparison between PeNGaRoo, SecretomeP and NonClasGP-Pred on independent test data.

To examine the performance of the NonClasGP-Pred predictor, we evaluated and compared it with two other state-of-the-art available predictors, namely PeNGaRoo and SecretomeP, which have been developed for predicting NCSPs of gram-positive bacteria. The independent test data were built on an independent dataset after removing the overlap sequence in the training dataset, thereby generating a more rigorous result and providing a fair comparison with existing tools. The results are presented in Fig. 6, where it can be seen that the NonClasGP-Pred clearly outperforms PeNGaRoo and SecretomeP in all five evaluation metrics on the independent test data, resulting in an ACC of 86.76 %, SN of 86.76 %, SP of 85.29 % and MCC of 73.56 % (Fig. 6a). In particular, the NonClasGP-Pred achieved an AUC of 0.9019 (Fig. 6b), which is 4.98 and 22.20 % higher than that of PeNGaRoo and SecretomeP, respectively. These results demonstrate that our ensemble predictor is significantly better than the existing prediction algorithms in the prediction of NCSPs.

### Webserver implementation

For convenience, we have established a publicly accessible webserver that implements our predictor, which can be freely accessed via http://lab.malab.cn/~wangchao/softwares/-NonClasGP/. Users will need to submit the query protein sequences in FASTA format. Then, by clicking on the Submit button, the predicted results will be presented on the screen and can be downloaded to a local computer.

## Conclusion

In this study, a new computational predictor, NonClasGP-Pred, was presented for NCSP prediction of gram-positive bacteria. First, ten balanced subdatasets were generated from the original imbalanced dataset, and ten sequence-based feature encodings were used to generate the feature space. Then, the feature representation ability was enhanced by SSF and subset-specific optimal feature combination strategies. Finally, an ensemble learning model was built to integrate all the subset-specific optimal models. Assessment of the independent test indicated that the proposed model outperformed state-of-the-art available toolkits. Through a series of analyses, we assumed that the improved performance by our predictor mainly contributed to feature selection, subset-specific model merging and ensemble strategies. A user-friendly web server that implements NonClasGP-Pred has been made available to maximize user convenience. NonClasGP-Pred is anticipated to be a useful bioinformatics tool for predicting the NCSPs of gram-positive bacteria and facilitating their functional understanding.

However, model performance resulting from only sequence-based features is limited to a certain degree. In future work, integrating sequence-based features with other evolutionary algorithms might be helpful for further performance improvement. Moreover, exploring more powerful machine learning algorithms, such as deep learning [48, 60–64] and unsupervised learning [65–67] is expected to effectively improve the predictive performance as well.

## Supplementary Data

Supplementary material 1
