# Supplementary material for: NonClasGP-Pred: robust and efficient prediction of non-classically secreted proteins by integrating subset-specific optimal models of imbalanced data
Source: Microb Genom. 2020 Nov 27;6(12):mgen000483. doi: 10.1099/mgen.0.000483 (PMC8116686; doi:10.1099/mgen.0.000483)
Supplement: Supplementary material 1 [file mgen-6-483-s001.pdf]

NonClasGP-Pred: Robust and efficient prediction of nonclassically secreted proteins by integrating subset-specific optimal models of imbalanced data

Chao Wang[1,#], Jin Wu[2,#], Lei Xu[3,*], Quan Zou[1,4,*]
1 Institute of Fundamental and Frontier Sciences, University of Electronic Science and Technology of China, Chengdu, China
2 School of Management, Shenzhen Polytechnic, Shenzhen, China
3 School of Electronic and Communication Engineering, Shenzhen Polytechnic, Shenzhen, China
4 Hainan Key Laboratory for Computational Science and Application, Hainan Normal University, Haikou, China
# equally contributed
*corresponding author: zouquan@nclab.net csleixu@szpt.edu.cn

Supplementary method

To build an accuracy and reliable bioinformatics tool, sufficient feature information should be incorporated into the model (Chen, et al., 2018; Liu, et al., 2019; Chen, et al., 2020; Wang, et al., 2020). In this study, 10 feature encoding algorithms were used for the protein sequence representing, including amino acid composition (AAC), composition of k-spaced amino acid pairs (CKSAAP), dipeptide composition (DPC), dipeptide deviation from expected mean (DDE), composition (CTDC), transition (CTDT), conjoint triad (CTriad), quasi-sequence-order (QSOrder), normalized Moreau-Broto (NMBroto) and pseudoamino acid composition (PAAC). For convenience, assume that a given protein sequence of N amino acid residues is denoted as $S=R_1R_2R_3R_4\ldots R_N$, where the i-th residue is represented as R_i. The detailed feature representation algorithm is explained in the following subsections.

AAC, DEP, CKSAAP and DDE

The AAC descriptor(Bhasin and Raghava, 2004; Liu, 2019) encodes the frequencies of all 20 amino acids in a protein sequence and is represented by a 20-D vector. The CKSAAP descriptor(Chen, et al., 2007) measures the frequency of any k residue-spaced amino acid pairs, the dimension of the this feature vector is 400×(k+1).

The DPC(Saravanan and Gautham, 2015) calculates the frequencies of all dipeptides in a sequence and is defined as:

$$\mathrm{D(r,s)} = \frac{N_{rs}}{N-1},\ r,s \in \{A,C,D\ldots Y\} \tag{1}$$

where N_{rs} is the number of dipeptides composed by r and s, which gives a 400-D vector. The DDE(Saravanan and Gautham, 2015), which also gives a 400-D vector, is computed as follows:

$$\mathrm{DDE(r,s)} = \frac{D_C(r,s) - T_m(r,s)}{\sqrt{T_V(r,s)}} \tag{2}$$

where $D_C(r,s)$ is calculated in a similar way as $D(r,s)$; $T_m(r,s)$, the theoretical mean, is calculated as:

$$T_M(r,s) = \frac{C_r}{C_N} \times \frac{C_s}{C_N} \tag{3}$$

where, for a dipeptide 'rs', C_r is the number of codons that code for the first amino acids and C_s is the number of codons that code for the second amino acids, and C_N is all possible codons, excepting stop codons. $T_V(r,s)$, the theoretical variance of the dipeptide 'rs', is defined as:

$$T_V(r,s) = \frac{T_M(r,s)(1-T_M(r,s)))}{N-1} \tag{4}$$

CTDC, CTDT and CTriad

The composition (C) and transition (T) features(Govindan and Nair, 2011) characterizes the amino acid distribution patterns or physicochemical property in a protein. Twenty amino acids are categorized into three groups according to their physicochemical property (supplementary Table S1). Taking the charge attribute for example, twenty amino acids are categorized into positive group (KR), neutral group (ANCQGHILMFPSTWYV) and negative group (DE). The three features of the composition descriptor represent the percentage of each group of residues in the protein sequence and is calculated as follows:

$$\mathrm{CTDC\ \ (r)} = \frac{N(r)}{N}, r \in \{postive, neutral, negative\} \tag{5}$$

where $N(r)$ is the number of amino acids of type r in a given sequence and N is the protein length.

The three features of the transition descriptor characterize the frequencies of three kinds of residue pairs. For example, two adjacent residues where a negative residue followed by a neutral residue or vice versa, it is calculated as follows:

$$\mathrm{CTDT(r,s)} = \frac{N(r,s)+N(s,r)}{N} \tag{6}$$

where$r,s \in \{(positive, neutral), (neutral, negative), (negative, positive)\}$, and $N(r,s)$ and $N(s,r)$ equal to the numbers of dipeptides composed by "rs" and "sr", respectively, in the protein sequence. Thirteen types of physicochemical properties (supplementary Table S1) are used for computing the features of CTDC and CTDT, which yield a 39-D vector for each feature.

CTriad(Shen, et al., 2007) characterizes the properties of one amino acid and its neighbors, where any three continuous amino acids were regarded as a single unit. Specifically, all 20 amino acids were categorized into seven groups based on their physicochemical properties. Then, all sets of the three successive amino acids (triad) within a given protein sequence were considered, and the triad frequencies were counted. Accordingly, CTriad is a 343-D vector and is defined as follows:

$$d_i = \frac{f_i - \min\{f_1, f_2, f_3, \ldots, f_{343}\}}{max\{f_1, f_2, f_3, \ldots, f_{343}\}}, i = 1,2,3, \ldots, 343 \tag{7}$$

where f_i denotes the frequency of the i-th triad that appears in the protein sequence.

QSOrder, NMBroto and PAAC

The first 20 features (Equation 8) of the QSOrder represents the amino acid frequency, and the remaining features characterize the sequence order based on the Schneider-Wrede physicochemical distance matrix(Schneider and Wrede, 1994) and the Grantham chemical distance matrix(Grantham, 1974) (Equation 9). It is defined as:

$$X_r = \frac{f_r}{\sum_{r=1}^{20} f_r + w \sum_{d=1}^{nlag} \tau_d}, r = 1, 2, 3, \ldots, 20 \quad (8)$$

$$X_d = \frac{w\tau_{d-20}}{\sum_{r=1}^{20} f_r + w \sum_{d=1}^{nlag} \tau_d}, d = 21, 2\ 2, 23, \ldots, nlag \quad (9)$$

$$\tau_d = \sum_{i=1}^{N-d} (d_{i,i+d})^2, d = 1, 2, 3, \ldots, nlag \quad (10)$$

where f_r is the normalized occurrence of amino acid type r and weighting factor w = 0.1; $d_{i,i+d}$ is the distance between the two amino acids at position i and i + d in protein sequence; *nlag* is the maximum value of the lag, N is the protein length .Accordingly, the descriptor dimension will be 40+2×nlag.

The NMBroto descriptor(Horne, 1988) is used to characterize the distribution of amino acid properties along the sequence. In this paper, eight amino acid indices are selected from the AAindex database (supplementary Table S2). The NMBroto is defined as follows:

$$\text{NMB} = \frac{AC(d)}{N-d}, d = 1, 2, \ldots, nlag \quad (11)$$

$$\text{AC(d)} = \sum_{i=1}^{N-d} P_i \times P_{i+d}, d = 1, 2, \ldots, nlag \quad (12)$$

where P_i and P_{i+d} are the related amino acid properties at positions i and i+d, respectively; d is the lag of the autocorrelation, nlag is the maximum value of the lag, N is the protein length, and the descriptor dimension is 8×nlag.

PAAC introduces a discrete model derived from the amino acid sequence to represent its sequence-order or pattern information. The PAAC descriptors(Chou, 2001, 2005) can be defined as follows:

Denoting the original hydrophobicity values of the 20 amino acids as H_1^O(i) (i = 1, 2, 3, …, 20). Similarly, the original hydrophobicity values and the original hydrophilicity values were denoted as H_2^O (i) and $M^O(i)$, respectively. They are transformed to the following quantities:

$$\begin{cases} H_1(i) = \dfrac{H_1^O(i) - \frac{1}{20}\sum_{i=1}^{20} H_1^O(i)}{\sqrt{\dfrac{\sum_{i=1}^{20}\left[H_1^O(i) - \frac{1}{20}\sum_{i=1}^{20} H_1^O(i)\right]^2}{20}}}, i = 1,2,3,\ldots,20 \\ H_2(i) = \dfrac{H_2^O(i) - \frac{1}{20}\sum_{i=1}^{20} H_2^O(i)}{\sqrt{\dfrac{\sum_{i=1}^{20}\left[H_2^O(i) - \frac{1}{20}\sum_{i=1}^{20} H_2^O(i)\right]^2}{20}}}, i = 1,2,3,\ldots,20 \\ M^O(i) = \dfrac{M^O(i) - \frac{1}{20}\sum_{i=1}^{20} M^O(i)}{\sqrt{\dfrac{\sum_{i=1}^{20}\left[M^O(i) - \frac{1}{20}\sum_{i=1}^{20} M^O(i)\right]^2}{20}}}, i = 1,2,3,\ldots,20 \end{cases} \quad (13)$$

$$\Theta(R_i, R_j) = \frac{1}{3}\{[H_1(R_i) - H_1(R_j)]^2 + [H_2(R_i) - H_2(R_j)]^2 + [M(R_i) - M(R_j)]^2\} \quad (14)$$

$$\theta_\lambda = \frac{1}{N-\lambda}\sum_{i=1}^{N-\lambda} \Theta(R_i, R_{i+\lambda}) \quad (15)$$

$$X_c = \frac{f_c}{\sum_{r=1}^{20} f_r + w\sum_{j=1}^{\lambda}\theta_j}, (1 \le c \le 20) \quad (16)$$

$$X_c = \frac{w\theta_{c-20}}{\sum_{r=1}^{20} f_r + w\sum_{j=1}^{\lambda}\theta_j}, (21 \le c \le 20 + \lambda) \quad (17)$$

where $H_k(R_i)$ denotes the kth property of the amino acid R_i in the amino acid property set, λ (λ < N) is an integer parameter that is chosen; f_c is the normalized occurrence of the amino acids, weighting factor w = 0.05, and N is the sequence length. The descriptor dimension will be 20+λ.

Supplementary Tables

Supplementary Table S1 Thirteen types of physicochemical properties that used for computing the features of CTDC and CTDT.

physicochemical properties	categorized groups		
Hydrophobicity_PRAM900101	Polar: RKEDQN	Neutral: GASTPHY	Hydrophobicity: CLVIMFW
Hydrophobicity_ARGP820101	Polar: QSTNGDE	Neutral: RAHCKMV	Hydrophobicity: LYPFIW
Hydrophobicity_ZIMJ680101	Polar: QNGSWTDERA	Neutral: HMCKV	Hydrophobicity: LPFYI
Hydrophobicity_PONP930101	Polar:KPDESNQT	Neutral: GRHA	Hydrophobicity:YMFWLCVI
Hydrophobicity_CASG920101	Polar:KDEQPSRNTG	Neutral: AHYMLV	Hydrophobicity: FIWC
Hydrophobicity_ENGD860101	Polar:RDKENQHYP	Neutral :SGTAW	Hydrophobicity: CVLIMF
Hydrophobicity_FASG890101	Polar: KERSQD	Neutral: NTPG	Hydrophobicity:AYHWVMFLIC

Normalized van der Waals volume	Volume range: 0-2.78GASTPD	Volume range: 2.95-94.0NVEQIL	Volume range: 4.03-8.08MHKFRYW
Polarity	Polarity value:4.9-6.2LIFWCMVY	Polarity value: 8.0-9.2PATGS	Polarity value: 10.4-13.0HQRKNED
Polarizability	Polarizability value: 0-1.08GASDT	Polarizability value:0.128-120.186GPNVEQIL	Polarizability value: 0.219-0.409KMHFRYW
Charge	Positive: KR	Neutral:ANCQGHILMFPST WYV	Negative: DE
Secondary structure	Helix:EALMQKR H	Strand: VIYCWFT	Coil: GNPSD
Solvent accessibility	Buried:ALFCGIV W	Exposed: PKQEND	Intermediate: MPSTHY

Supplementary Table S2 Amino acid indices selected from the AAindex database used for NMBroto descriptor.

**Amino acid indices**	**Description**
CIDH920105	Normalized average hydrophobicity scales
BHAR880101	Average flexibility indices
CHAM820101	Polarizability parameter
CHAM820102	Free energy of solution in water, kcal/mole
CHOC760101	Residue accessible surface area in tripeptide
BIGC670101	Residue volume
CHAM810101	Steric parameter
DAYM780201	Relative mutability

https://www.genome.jp/aaindex/

Supplementary Table S3 Preliminary experiment results of feature combination.

Feature combination among the ten feature subsets using an exhaustive searching. We evaluated all possible 1023 models for each of the ten training dataset TD1, the maximum value of accuracy and the related value on independent test data are listed a follows.

c_{10}^{1}	max acc of 10-fold CV: 0.9362<br>independent_test_score: 0.8529	c_{10}^{6}	max acc of 10-fold CV: 0.9400<br>independent_test_score: 0.7941
c_{10}^{2}	max acc of 10-fold CV: 0.9398<br>independent_test_score: 0.8382	c_{10}^{7}	max acc of 10-fold CV: 0.9400<br>independent_test_score: 0.8088
c_{10}^{3}	max acc of 10-fold CV: 0.9398<br>independent_test_score: 0.7941	c_{10}^{8}	max acc of 10-fold CV: 0.9400<br>independent_test_score: 0.7941
c_{10}^{4}	max acc of 10-fold CV: 0.9431<br>independent_test_score: 0.7941	c_{10}^{9}	max acc of 10-fold CV: 0.9364<br>independent_test_score: 0.8088
c_{10}^{5}	max acc of 10-fold CV: 0.9433<br>independent_test_score: 0.7941	c_{10}^{10}	max acc of 10-fold CV: 0.9364<br>independent_test_score: 0.7647

Supplementary Table S4 Performance comparison between the models built on individual training subsets and the ensemble model by 10-fold cross validation.

**Subdataset**	**ACC**	**SN**	**SP**	**MCC**	**AUC**
TD_1	0.857882	0.821905	0.892857	0.7217	0.9142
TD_2	0.918596	0.942857	**0.89381**	0.842793	0.9660
TD_3	0.893103	0.921429	0.864286	0.792923	0.9527
TD_4	0.857759	0.9	0.815238	0.723731	0.9253
TD_5	0.858128	0.88619	0.830952	0.727112	0.9407
TD_6	0.843719	0.857619	0.829524	0.693192	0.9111
TD_7	0.857512	0.85	0.864286	0.72676	0.9281
TD_8	0.83633	0.864762	0.808095	0.67739	0.9180
TD_9	0.871798	0.843333	0.9	0.75071	0.9390
TD_10	0.882759	0.9	0.865714	0.771259	0.9405
Ensemble	**0.932266**	**1**	0.890123	**0.876823**	**0.9975**

References

Bhasin, M. and Raghava, G.P.S. Classification of nuclear receptors based on amino acid composition and dipeptide

composition. *J. Biol. Chem.* 2004;279(22):23262-23266.
Chen, K., Kurgan, L. and Rahbari, M. Prediction of protein crystallization using collocation of amino acid pairs. *Biochem. Biophys. Res. Commun.* 2007;355(3):764-769.
Chen, Z.*, et al.* iFeature: a Python package and web server for features extraction and selection from protein and peptide sequences. *Bioinformatics* 2018;34(14):2499-2502.
Chen, Z.*, et al.* iLearn: an integrated platform and meta-learner for feature engineering, machine-learning analysis and modeling of DNA, RNA and protein sequence data. *Brief Bioinform* 2020;21(3):1047-1057.
Chou, K.C. Prediction of protein cellular attributes using pseudo-amino acid composition. *Proteins* 2001;43(3):246-255.
Chou, K.C. Using amphiphilic pseudo amino acid composition to predict enzyme subfamily classes. *Bioinformatics* 2005;21(1):10-19.
Govindan, G. and Nair, A.S. Composition, transition and distribution (ctd) - a dynamic feature for predictions based on hierarchical structure of cellular sorting. In: Negi, A.*, et al.*, editors, *2011 Annual Ieee India Conference*. New York: Ieee; 2011.
Grantham, R. Amino acid difference formula to help explain protein evolution. *Science (New York, N.Y.)* 1974;185(4154):862-864.
Horne, D.S. Prediction of protein helix content from an autocorrelation analysis of sequence hydrophobicities. *Biopolymers* 1988;27(3):451-477.
Liu, B. BioSeq-Analysis: a platform for DNA, RNA, and protein sequence analysis based on machine learning approaches. *Briefings in Bioinformatics* 2019;20(4):1280-1294.
Liu, B., Gao, X. and Zhang, H. BioSeq-Analysis2.0: an updated platform for analyzing DNA, RNA, and protein sequences at sequence level and residue level based on machine learning approaches. *Nucleic Acids Research* 2019;47(20):e127.
Saravanan, V. and Gautham, N. Harnessing computational biology for exact linear b-cell epitope prediction: A novel amino acid composition-based feature descriptor. *Omics* 2015;19(10):648-658.
Schneider, G. and Wrede, P. The rational design of amino-acid-sequences by artificial neural networks and simulated molecular evolution - de-novo design of an idealized leader peptidase cleavage site. *Biophys. J.* 1994;66(2):335-344.
Shen, J.W.*, et al.* Predictina protein-protein interactions based only on sequences information. *Proc. Natl. Acad. Sci. U. S. A.* 2007;104(11):4337-4341.
Wang, M.*, et al.* Prediction of Extracellular Matrix Proteins by Fusing Multiple Feature Information, Elastic Net, and Random Forest Algorithm. *Mathematics* 2020;8(2):169.
